# Variability in commercially available deformable image registration: A multi‐institution analysis using virtual head and neck phantoms

**DOI:** 10.1002/acm2.13242

**Published:** 2021-03-30

**Authors:** Alex Kubli, Jason Pukala, Amish P. Shah, Patrick Kelly, Katja M. Langen, Frank J. Bova, Rafael R. Mañon, Sanford L. Meeks

**Affiliations:** ^1^ Department of Radiation Oncology Thomas Jefferson University Hospital Philadelphia PA USA; ^2^ Department of Radiation Oncology Orlando Health Cancer Institute Orlando FL USA; ^3^ Department of Radiation Oncology Emory University Atlanta GA USA; ^4^ Department of Neurosurgery University of Florida Gainesville FL USA

**Keywords:** deformable, error, image, registration, virtual phantoms

## Abstract

**Purpose:**

The purpose of this study was to evaluate the performance of three common deformable image registration (DIR) packages across algorithms and institutions.

**Methods and Materials:**

The Deformable Image Registration Evaluation Project (DIREP) provides ten virtual phantoms derived from computed tomography (CT) datasets of head‐and‐neck cancer patients over a single treatment course. Using the DIREP phantoms, DIR results from 35 institutions were submitted using either Velocity, MIM, or Eclipse. Submitted deformation vector fields (DVFs) were compared to ground‐truth DVFs to calculate target registration error (TRE) for six regions of interest (ROIs). Statistical analysis was performed to determine the variability between each DIR software package and the variability of users within each algorithm.

**Results:**

Overall mean TRE was 2.04 ± 0.35 mm for Velocity, 1.10 ± 0.29 mm for MIM, and 2.35 ± 0.15 mm for Eclipse. The MIM mean TRE was significantly different than both Velocity and Eclipse for all ROIs. Velocity and Eclipse mean TREs were not significantly different except for when evaluating the registration of the cord or mandible. Significant differences between institutions were found for the MIM and Velocity platforms. However, these differences could be explained by variations in Velocity DIR parameters and MIM software versions.

**Conclusions:**

Average TRE was shown to be <3 mm for all three software platforms. However, maximum errors could be larger than 2 cm indicating that care should be exercised when using DIR. While MIM performed statistically better than the other packages, all evaluated algorithms had an average TRE better than the largest voxel dimension. For the phantoms studied here, significant differences between algorithm users were minimal suggesting that the algorithm used may have more impact on DIR accuracy than the particular registration technique employed. A significant difference in TRE was discovered between MIM versions showing that DIR QA should be performed after software upgrades as recommended by TG‐132.

## 1 INTRODUCTION

When treating a patient, anatomical changes that occur over the treatment course can result in meaningful effects on radiation dose delivery.^1^, ^2^ To quantify the impact of these changes, it is important to identify corresponding physical points between multiple images of the same patient. Deformable image registration (DIR) has become available in many commercial treatment planning and contouring systems for this purpose. This is particularly useful to propagate contours from one image to another^3^ or accumulate dose across a treatment.^4^ Understanding the accuracy of DIR is a critical duty for the clinician, as the DIR accuracy may influence clinical decisions. For example, the DIR accuracy for contour propagation may be less stringent, as long as the propagated contours are reviewed and corrected, than the accuracy required for deformable dose accumulation where decisions may be made concerning organ‐at‐risk (OAR) tolerances. This study aims to further the comprehension of DIR accuracy for typical head and neck cancer patients over the treatment course.

Deformable image registration is a non‐affine process that uses mathematical models to deform one image to match another. Unlike rigid or affine registration, each voxel in DIR is assigned a deformation vector that may be only loosely dependent on neighboring voxels. The resulting deformed image is characterized by its deformation vector field (DVF), vector matrices which define the relationship between the original and deformed images. Due to the high number of degrees of freedom present in DIR algorithms, the DVF output from an algorithm may result in an image deformation that is not biologically or geometrically plausible.

Because of the freedom with which DIR may deform an image, there have been research efforts to validate the use of DIR in radiation therapy. Ground‐truth models with a known DVF relating pre‐ and post‐deformation images, or with phantoms containing known landmarks, are often used for this purpose.^5^, ^6^, ^7^, ^8^, ^9^, ^10^ Each of these ground‐truth models provides a framework that can help the clinical physicist validate the DIR implemented in their clinic. This study uses virtual phantoms provided by the Deformable Image Registration Evaluation Project (DIREP).^10^ Deformable Image Registration Evaluation Project created publicly available phantoms based on computed tomography (CT) data from head and neck cancer patients. These phantoms provide a clinically based ground‐truth model that encompasses the anatomical changes that occur over the course of a typical treatment.

As an additional tool to validate DIR accuracy, AAPM’s Task Group 132 (TG‐132) report provides a framework to commission and provides QA of DIR output.^11^ The report provides several digital deformable phantoms for testing DIR along with their recommended tolerances. When using DIR, TG‐132 does recommend that relevant boundaries and anatomical features in the registered images be within 1–2 voxels, and that any additional error should feed into planning margins. However, it does not provide information on how the digital phantoms might compare to clinical cases or site‐specific accuracy expectations.

Several studies have quantified DIR accuracy from different commercial or research algorithms, using data submitted by multiple institutions. This has been done using either contour‐based^12^, ^13^ or landmark‐based^14^, ^15^ analysis. Contour‐based methods can be subjective, as they introduce variability based on the observer drawing the contours and contain little information about the accuracy of voxels within the contour. Landmark‐based methods provide accuracy information in the vicinity of the landmark, but are limited to those regions. In both cases, a contour or landmark that is easy for a human to identify may also be easy for an algorithm to identify. This may produce biased accuracy data. Comparatively, the DIREP model assesses deformation accuracy for each voxel by comparing the entire registration DVF to the ground‐truth DVF. Additionally, the previous studies referenced have been limited by sample size, with a maximum of 14 institutions submitting results for commercially available algorithms.

To establish a benchmark for DIR accuracy, several commercial algorithms were previously tested with the DIREP phantoms.^16^ Following the benchmarking study, 35 institutions have submitted DIREP registrations for the complete phantom set. The aim of this work is to analyze the data submitted from these institutions using the DIREP ground‐truth model in order to characterize the inter‐algorithm and inter‐institutional variability of three commercial DIR software packages: Velocity (Varian Medical Systems, Palo Alto, CA), MIM (MIM Software Inc., Cleveland, OH), and Eclipse (Varian Medical Systems, Palo Alto, CA). This is done to provide the clinical physicist with insight that can assist in implementing DIR clinically, and to enhance the understanding of the inherent accuracy and limitations of these DIR algorithms. Additionally, this study aims to use these results to augment the expectations set by the recommendations of TG‐132.

## 2 METHODS

### 2.A Ground‐truth model

**Fig. 1:**
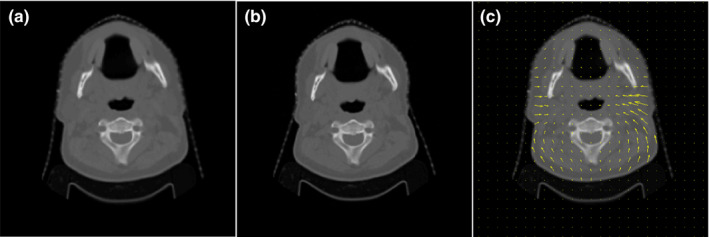
Virtual phantom example. (a) Start‐of‐treatment image. (b) End‐of‐treatment image. (c) Start‐of‐treatment image with the ground‐truth DVF overlaid.

Deformable Image Registration Evaluation Project provides ten virtual phantoms, based on CT images taken at the start of treatment (SOT) and near the end of treatment (EOT) for ten patients treated for head and neck cancer. All of the virtual phantom datasets had an in‐plane resolution of 0.97–1.37 mm and a slice thickness of 3 mm.^10^ A biomechanical algorithm^17^, ^18^ and a thin‐plate splines algorithm^19^ were used to create an anatomically representative pair of images where the underlying “true” deformation field was known. The thin‐plate splines algorithm was available as a deformation tool within the ImSimQA software package (Oncology Systems Limited, Shrewsbury, Shropshire, UK). In all, these tools allowed for the modeling of the following anatomical changes: head rotation and translation, mandible rotation, spine flexion, shoulder movement, hyoid movement, tumor/node shrinkage, weight loss, and parotid shrinkage. Physician‐drawn brainstem, spinal cord, mandible, left parotid, and right parotid contours are included in these phantoms to allow for the analysis of those structures. Figure 1 shows an example of one of the DIREP phantoms along with its associated ground‐truth DVF.

### 2.B Basic DIR evaluation

To test the accuracy of a given DIR process, each EOT phantom is registered to its associated SOT phantom, as would be performed for dose accumulation. The DVFs created by each of these registrations can then be directly compared to the ground‐truth DVFs provided by the DIREP phantoms. Complete datasets were submitted by 35 institutions. The algorithms used by the submitting institutions are listed in Table 1. The companies behind these DIR algorithms do not typically publish detailed information on their algorithms to protect intellectual property. Aside from the information given in Table 1, these algorithms will be treated as a “black box” for the purposes of this study.

**Table 1:** Details of the DIR algorithms evaluated in this study.

Software	Versions	DIR algorithm	Algorithm type	Number of institutions
Velocity	3.1 to 3.2.1	Deformable multi‐pass or extended deformable multi‐pass	Multiresolution B‐spline	16
MIM	6.2.2 to 6.7.7	VoxAlign	Constrained, intensity‐based, free‐form	13
Eclipse	11 to 13.7	SmartAdapt	Accelerated demons	6

Using target registration error (TRE) as a figure of merit, the DVFs submitted by each institution were compared to the ground‐truth DVFs for all ten phantoms. Registration accuracy was evaluated for six regions of interest (ROIs): brainstem, spinal cord, mandible, left parotid, right parotid, and external contour. From this, the TRE from the ground truth for each voxel in each ROI was calculated. These values were summarized into a mean TRE across each region for each phantom. The maximum deviation in each region was also recorded. This information was averaged across all ten phantoms in order to calculate a set of summary statistics representing the overall accuracy of the deformable registration for each institution.

### 2.C Statistical analysis

#### 2.C.1 Inter‐algorithm variability

To determine inter‐algorithm variability, the summary statistics for each institution were grouped by the registration software used for the deformation. A Welch’s *t*‐test was performed in order to compare the mean TRE for each combination of DIR algorithms, for each contoured region. This was done to test the null hypothesis that any two algorithms have the same mean TRE.

#### 2.C.2 Inter‐institutional variability

In order to evaluate the variability within each algorithm, the institutional data were again grouped by the DIR algorithm used. Because the summary statistics for an institution are averaged across the 10 DIREP phantoms, an ANOVA was performed to test the null hypothesis that the average TRE within an algorithm for each contour was not institutionally dependent.

## 3 RESULTS

### 3.A Summary statistics

Table 2 shows the summary statistics for all institutions submitting data using Velocity, MIM, and Eclipse. On average, MIM performed the tests with the smallest registration error, with average TRE values consistently smaller than the other two algorithms. However, the maximum error for MIM is greater than the other two algorithms in areas that tend to fluctuate in overall volume throughout the course of a treatment, in particular with both parotid contours.

**Table 2:** Summary statistics for all institutions that submitted data using Velocity, MIM, and Eclipse. Errors shown are ±standard error of the mean.

Region	Velocity (N = 16)		MIM (N = 13)		Eclipse (N = 6)	
	Mean (mm)	Max (mm)	Mean (mm)	Max (mm)	Mean (mm)	Max (mm)
Brainstem	1.23 ± 0.23	4.8	0.42 ± 0.10	2.4	1.15 ± 0.10	7.3
Cord	1.40 ± 0.24	12.2	0.38 ± 0.15	2.7	1.12 ± 0.04	4.6
Mandible	1.34 ± 0.23	9.0	0.68 ± 0.24	8.2	1.97 ± 0.08	9.4
Left parotid	1.92 ± 0.21	10.8	1.12 ± 0.11	13.1	1.92 ± 0.12	11.0
Right parotid	1.59 ± 0.26	13.1	1.23 ± 0.28	26.9	1.75 ± 0.10	8.9
External	2.04 ± 0.35	40.7	1.10 ± 0.29	34.7	2.35 ± 0.15	25.0

**Fig. 2:**
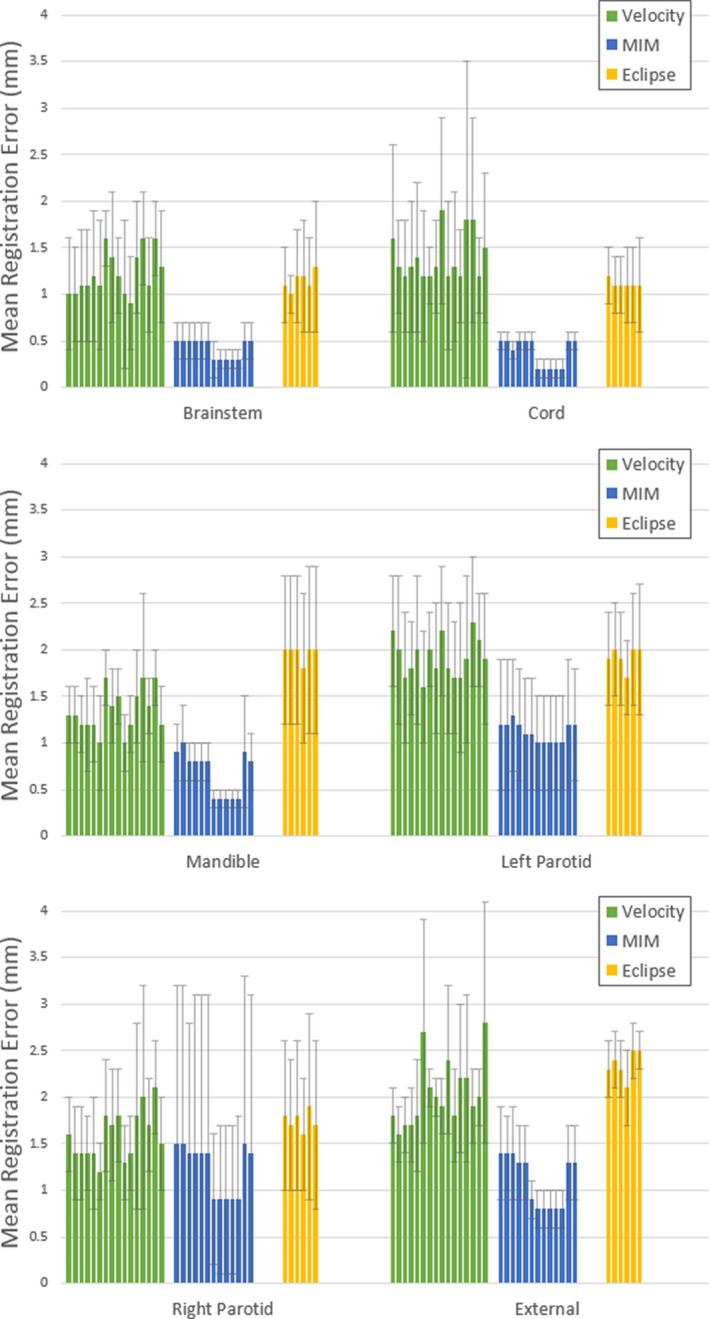
Mean TRE for each institution in this study. The error bars represent one standard deviation of the mean registration error for each of the ten phantoms. Note that the Y‐axis scale is 4 mm and that the mean TRE difference between institutions is typically <1 mm.

The data can be seen in more detail, separated by institution, in Fig. 2. Upon review of these data, it can be seen that the institutions using Velocity and Eclipse showed similar results across all contours. The exceptions to this are the cord, where Eclipse consistently outperformed Velocity, and for the mandible, where Eclipse consistently performed worse than Velocity. For all other contours, some of the Velocity institutions performed better than some of the Eclipse institutions and vice versa. The MIM institutions, however, exhibited a lower mean TRE than all of the Velocity and Eclipse institutions for all of the contours except for the right parotid. Within the MIM results, there appears to be a bimodal distribution in that the same institutions consistently yielded a lower mean TRE across all contours.

### 3.B Inter‐algorithm variability

Table 3 shows the full results of a Welch’s *t*‐test applied to the average TRE for each combination of registration algorithms, separated by region. In order to adjust the false‐positive rate to account for performing 18 separate tests, an initial alpha threshold of 0.05 was corrected using a Bonferroni correction to an adjusted value of 0.0028. From Table 3, it can be seen that the mean TRE using MIM is significantly different than the mean TRE using either Velocity or Eclipse. For the brainstem, both parotids, and external contours, there was a failure to reject the null hypothesis that the mean TRE between Velocity and Eclipse is the same. This suggests that for the DIREP head and neck phantoms, both Velocity and Eclipse performed similarly on average in most cases. These results are consistent with the institution‐specific results contained in Fig. 2.

**Table 3:** Results of Welch’s *t*‐test to compare mean TREs across algorithms. Tests marked with an asterisk (*) indicate a significant difference in mean TRE.

Algorithm 1	Algorithm 2	Region	*P*‐value
Velocity	MIM	Brainstem	<0.001*
Velocity	Eclipse	Brainstem	0.312
MIM	Eclipse	Brainstem	<0.001*
Velocity	MIM	Cord	<0.001*
Velocity	Eclipse	Cord	<0.001*
MIM	Eclipse	Cord	<0.001*
Velocity	MIM	Mandible	<0.001*
Velocity	Eclipse	Mandible	<0.001*
MIM	Eclipse	Mandible	<0.001*
Velocity	MIM	Parotid L	<0.001*
Velocity	Eclipse	Parotid L	0.977
MIM	Eclipse	Parotid L	<0.001*
Velocity	MIM	Parotid R	0.001*
Velocity	Eclipse	Parotid R	0.058
MIM	Eclipse	Parotid R	<0.001*
Velocity	MIM	External	<0.001*
Velocity	Eclipse	External	0.009
MIM	Eclipse	External	<0.001*

### 3.C Inter‐institutional variability

Tables 4 shows the results of one‐way ANOVAs performed to test the null hypothesis that the mean TRE for a given algorithm, within a given region, is not institutionally dependent. Because there were 18 comparisons made, the significance threshold of 0.05 was altered with a Bonferroni correction to an adjusted value of 0.0028. For most cases, algorithm performance is not significantly user dependent. There is a significant institutional impact on the mandible and external contour registrations when using Velocity, but overall user dependence on mean TRE is minimal; these differences account for a sub‐mm change in registration accuracy, which is likely not clinically significant. When all of the MIM submissions are grouped together, the mean TRE for the MIM data is user dependent in all regions except for the parotids. This is not surprising, given the bimodal distribution shown in Fig. 2.

**Table 4:** Results of one‐way ANOVAs performed on mean TRE for each region. Regions that show a significant institutional dependence are indicated by an asterisk (*).

Region	Magnitude (mm)	F‐value	*P*‐value
Velocity			
Brainstem	1.23	1.62	0.074
Cord	1.40	0.873	0.596
Mandible	1.34	2.92	< 0.001*
Left parotid	1.92	0.928	0.535
Right parotid	1.59	1.87	0.031
External	2.04	2.93	< 0.001*
MIM			
Brainstem	0.42	5.56	< 0.001*
Cord	0.38	31.7	< 0.001*
Mandible	0.68	12.4	< 0.001*
Left parotid	1.12	0.526	0.894
Right parotid	1.23	0.650	0.795
External	1.10	13.8	< 0.001*
Eclipse			
Brainstem	1.15	0.426	0.829
Cord	1.12	0.119	0.988
Mandible	1.97	0.096	0.993
Left parotid	1.92	0.466	0.800
Right parotid	1.75	0.168	0.974
External	2.35	2.46	0.044

Although MIM initially showed an overall user dependence on registration accuracy, there appeared to be a difference between the mean TRE produced by MIM versions 6.5 and prior, when compared to MIM versions after 6.6. Table 5 shows the mean and maximum TRE for each region for MIM data grouped by versions. Once data were grouped in this way, Welch’s t‐tests were performed in order to test for a significant difference in mean TRE through this grouping. From these data, it is clear that there is a notable improvement in mean TRE after version 6.6. The maximum error is also notably lower for MIM versions after 6.6, especially for the mandible, parotid, and external contours. Beyond this, the variability found both pre‐ and post‐MIM version 6.6 is lower than the overall variability found in MIM when these data were grouped together; the true variability in MIM output is lower than what was shown in Table 2.

**Table 5:** Registration error and Welch’s *t*‐test results for MIM before and after version 6.6. The *t*‐test compared mean TRE across version grouping. Errors shown are ±standard error of the mean. Asterisk (*) indicate significant values.

	Pre‐6.6 (N = 8)		Post‐6.6 (N = 5)		*P*‐value
	Mean (mm)	Max (mm)	Mean (mm)	Max (mm)	
Brainstem	0.45 ± 0.01	2.4	0.31 ± 0.00	1.4	<0.001*
Cord	0.47 ± 0.03	2.7	0.23 ± 0.00	2.1	<0.001*
Mandible	0.87 ± 0.08	8.2	0.41 ± 0.00	5.8	<0.001*
Left parotid	1.20 ± 0.05	13.1	0.97 ± 0.01	10.7	<0.001*
Right parotid	1.46 ± 0.02	26.9	0.92 ± 0.01	10.9	<0.001*
External	1.36 ± 0.04	34.7	0.79 ± 0.01	26.3	<0.001*

One‐way ANOVAs, shown in Table 6, were performed in order to test for user variability both before and after this change in version. From this, it was found that there was no user dependence on registration accuracy for pre‐6.6 and post‐6.6 registrations when treated as separate groups. Consequently, the user dependence found with MIM is likely the result of a difference in algorithm between MIM versions, and not a user dependence on the software.

**Table 6:** One‐way ANOVA results for MIM mean TRE before and after version 6.6.

Region	Magnitude (mm)	F‐value	*P*‐value
MIM pre‐6.6			
Brainstem	0.50	0.002	1.00
Cord	0.49	0.033	0.999
Mandible	0.85	0.087	0.999
Left parotid	1.19	0.015	0.999
Right parotid	1.44	0.003	1.00
External	1.34	0.008	1.00
MIM post‐6.6			
Brainstem	0.30	0.002	0.999
Cord	0.20	0.000	1.00
Mandible	0.40	0.001	0.999
Left parotid	1.00	0.001	0.999
Right parotid	0.90	0.001	0.999
External	0.82	0.004	0.999

## 4 DISCUSSION

In all, the summary statistics compiled here reflect 350 individual registrations. While the maximum errors on the order of 25–40.7 mm are concerning, the mean errors (ranging from 0.38 to 2.35 mm) were less than the largest voxel dimension (3 mm). Although MIM consistently produced the smallest errors for this dataset, to our knowledge, there are no aspects of the phantoms that would have biased the results toward that algorithm. In fact, the DIREP phantoms were created using multiple algorithms and human interaction in an attempt to eliminate bias toward any algorithm.^10^

The outlier in the MIM data appears in the right parotid results. For eight of the MIM institutions, the standard deviation of the registration error for the right parotid contour was much greater than was seen in the other ROIs. This was the result of a failure of the MIM algorithm within the contour of the right parotid for one of the DIREP phantoms. As shown in a previous study,^16^ MIM was unable to reproduce the correct registration of the right parotid for Phantom 9. This investigation found mean and maximum registration errors similar to the previous study (greater than 6 and 20 mm, respectively) for the right parotid of Phantom 9 prior to MIM version 6.6. With version 6.6, the mean and maximum TRE for the right parotid of Phantom 9 were reduced to just over 3 and 10.9 mm, respectively. This finding emphasizes the changes that may occur, in this case improvements, as updates are made to DIR algorithms.

When comparing algorithm performance, Table 3 shows that the difference between Velocity and Eclipse was not significant for most ROIs. The exceptions are for the cord and mandible where one algorithm consistently performed better than the other. This is consistent with the data observed in Fig. 2. Table 3 also shows that the MIM average TRE was significantly different than both Velocity and Eclipse for all of the evaluated ROIs. However, these differences were typically on the order of about 1 mm. Such small differences may not be clinically significant when considering contour propagation, but may be significant when reviewing dose accumulation, especially in high‐dose gradient regions.^16^

A surprising finding was that, on average, the differences in TRE between institutions using the same software were not statistically significant when accounting for the change in MIM versions as shown in Tables 4, 5, 6. The lone exceptions to this were the mandible and external ROIs for institutions using Velocity. Upon further investigation, it was discovered that the difference in registration error between institutions for the mandible was most likely due to the use of the Deformable Multi Pass (DMP) algorithm versus the Extended Deformable Multi Pass (EDMP) algorithm. Unfortunately, further analysis comparing the DMP to the EDMP algorithm was prohibited because not all sites reported which was used at the time of submission. Within Velocity, a user‐defined 3D ROI may be used to define the region to be deformed. Any part of the dataset outside of this ROI is not deformed. A subset of the Velocity users elected to define an ROI that excluded the patients’ shoulders in an effort to improve the DIR in the rest of the dataset. While an improvement in the registration of the anatomical ROIs was not obvious when using the DIR‐limiting ROI, the shoulder regions were misregistered resulting in large DIR errors in those regions compared to the ground truth. This is the reason for the statistically significant difference of the external ROI between Velocity institutions. The lack of significant differences between users of a particular DIR platform suggests that, at least for the displacements seen in these virtual phantoms representing head and neck cancer patients over a typical course of treatment, the choice of algorithm may be more impactful than the registration technique employed by trained users.

Other multi‐institution studies that have evaluated commercial algorithms include those published by Loi et al^12^, ^13^ and Kadoya et al.^14^ The first study published by Loi et al assessed submissions from 13 centers using six different commercial DIR platforms, including RayStation (RaySearch Laboratories, Stockholm, Sweden), MIM, Velocity, Eclipse, Mirada XD (Mirada Medical Ltd, Oxford, UK), and ABAS (Elekta AB, Stockholm, Sweden).^12^ That investigation evaluated three virtual phantoms representing the head and neck region, pelvis, and thorax. The second Loi et al study added an institution but primarily investigated the performance of DIR algorithms for multimodal (cone beam CT and megavoltage CT) registration using head and neck virtual phantoms.^13^ While it is difficult to directly compare those studies to this work because only contour‐matching metrics were reported (e.g., Dice similarity coefficient and mean distance to conformity), both studies found significant differences between the various platforms used but not between users of the same algorithm. This is similar to what we have reported.

Kadoya et al evaluated DIR performance using landmark analysis of thoracic 4D CT datasets.^14^ Eleven institutions submitted data for RayStation, MIM, and Velocity. The mean registration error found in that work was 3.28 mm for RayStation, 3.29 mm for MIM, and 5.01 mm for Velocity. While the errors seen in that study are higher than those reported here, that is likely the result of the greater anatomical displacement seen in 4D thoracic datasets than in head and neck cancer patients. In fact, Kadoya et al found that the cases that were tested with the largest displacement between peak inhale and peak exhale exhibited the largest registration error with the highest standard deviation. The Kadoya study also found moderate variation among institutions using the same DIR software, contrary to this work. This suggests that the technique used for registration or the proficiency of the user may have more of an influence on the DIR error for datasets with greater anatomical variation.

TG‐132 recommends that DIR platforms be tested during commissioning, annual QA, and upon upgrade, and provides digital phantoms for this purpose.^11^ The recommended tolerance for one of the phantom datasets is that 95% of voxels within the phantom be within 2 mm with a maximum error less than 5 mm. While many of the individual OARs examined in this study may meet that criteria, the entire phantom does not as seen in the summary statistics for the external contour in Table 2. In other words, clinicians should not assume that the results achieved during testing are indicative of the results achievable in all situations or, presumably, with complex clinical datasets. TG‐132 further recommends that patient‐specific deformable registration QA should verify that relevant anatomic boundaries and features are within 1–2 voxels, and that any additional error should feed into margins. For the OARs studied, this work could assist clinicians in determining the minimum margins required when using DIR for dosimetric evaluation.

The primary limitation of this study is that the findings are likely only applicable to the type of patients represented by the virtual phantoms, that is head and neck cancer patients over a single course of treatment. Additionally, while a variety of patients were chosen for creation of the virtual phantoms, only ten phantoms were created and evaluated which may not be representative of the anatomic changes seen in the entire population of head and neck radiotherapy patients. These results should not be extrapolated to other sites or situations (e.g., retreatments) without further research to confirm their validity. Furthermore, while the authors found no significant difference between users of the same DIR algorithms in this study, the use of advanced tools was not investigated. As new DIR tools are developed by vendors, such as the ability to refine a registration or contour‐guided DIR, these tools may provide more options and differentiation between users.

## 5 CONCLUSIONS

Three hundred and fifty registrations from 35 institutions were evaluated for DIR accuracy in this study. While it was shown that the average error was <3 mm for all three software platforms, care should be exercised when using DIR because localized or maximum errors can be much greater. The authors found that one algorithm performed statistically better than the others, but that all algorithms were typically more accurate than the largest voxel dimension. For the relatively small displacements between registered images studied here, no significant inter‐institutional difference was found between users of the same algorithm. This suggests that, for head and neck DIR within a treatment course, the algorithm used may have more of an impact on registration accuracy than a trained user’s DIR technique. A significant difference between versions of one of the algorithms was reported. This finding supports the TG‐132 recommendation that registration algorithms should be tested upon upgrade. Unfortunately, the current DIR testing options are often time‐consuming and limited to academic centers. To enable more frequent testing and the use of appropriate DIR margins, vendors should provide analysis tools to simplify testing for various sites and situations.

## CONFLICT OF INTEREST

Dr. Pukala reports grants from Accuray, Inc., outside the submitted work. Dr. Langen reports personal fees from Varian Medical, outside the submitted work.

## AUTHOR CONTRIBUTIONS

All listed authors made substantial contributions to this work, assisted in the drafting or review of this work, will have the opportunity to approve the final version to be published (if revisions are required), and agree to be accountable for all aspects of the work.
